# The Epidemiological Pattern and Co-infection of Influenza A and B by Surveillance Network From 2009 to 2014 in Anhui Province, China

**DOI:** 10.3389/fpubh.2022.825645

**Published:** 2022-02-24

**Authors:** Jun He, Sai Hou, Yue Chen, Jun-Ling Yu, Qing-Qing Chen, Lan He, Jiang Liu, Lei Gong, Xin-Er Huang, Jia-Bing Wu, Hai-Feng Pan, Rong-Bao Gao

**Affiliations:** ^1^Microbiological Laboratory, Anhui Provincial Center for Disease Control and Prevention, Hefei, China; ^2^Microbiological Laboratory, Public Health Research Institute of Anhui Province, Hefei, China; ^3^Department of Epidemiology and Biostatistics, School of Public Health, Anhui Medical University, Hefei, China; ^4^Inflammation and Immune Mediated Diseases Laboratory of Anhui Province, Hefei, China; ^5^Huainan City Center for Disease Control and Prevention, Huainan, China; ^6^Department of Health Inspection and Quarantine, School of Public Health, Anhui Medical University, Hefei, China; ^7^NHC Key Laboratory of Biosafety, NHC Key Laboratory of Medical Virology and Viral Diseases, National Institute for Viral Disease Control and Prevention, Chinese Center for Disease Control and Prevention, Beijing, China

**Keywords:** influenza-like illness, H1N1pdm09, H3N2, influenza B, co-infection

## Abstract

Influenza-like illness (ILI) is one of the most important public health problems globally, causing an enormous disease burden. Influenza infections are the most common cause of ILI. Bacterial and virus co-infection is common yet the data of co-infection with influenza A and B viruses are scarce. To identify the epidemiological patterns of and co-infection of influenza A and B in Anhui province, China, we analyzed the surveillance data of 5 years from 2009 to 2014 collected by the Chinese National influenzas network. The results showed that the weekly ratio of ILI was 3.96 ± 1.9% (95% CI 3.73–4.2%) in outpatients and the highest affected population was children under 5 years old. The epidemic of influenza viruses was highest during 2009–2010. For the other 4 surveillance years, school-aged people (5–14 years) were the most highly affected population. Influenza B and H3N2 viruses were more prevalent than H1N1pdm09 virus after 2010. In addition, a significant co-circulation of influenza A (H1N1pdm09 and H3N2) and influenza B virus was detected with 0.057% PCR positive rate during 2009–2014 in Eastern China, yet isolated only in pediatric patients. Our data reveals school-aged population would be the main vulnerable population and a distinct seasonality for influenza. In addition, the co-infection of influenza A and B were found in Anhui Province, China. Ongoing surveillance is critical to understand the seasonality variation and make evidence-based vaccination recommendations. Information on the epidemiological patterns and co-infections of influenza A and B can help us to implement different strategies for selecting vaccine formulations and monitoring new emerging influenza strains. In addition, the identification of the susceptible population can help us to develop more precise protection measures.

## Introduction

Influenza virus, one of the most important respiratory viruses presenting globally annual seasonal epidemics, causes fatal respiratory diseases to humans. The estimated number of global annual influenza-associated respiratory deaths were 291,243–645,832 per year during the 1999–2015 period, and 9,243–105,690 deaths occur annually among children younger than 5 years (1). The mortality related to influenza virus infections adhere to a cyclic pattern fluctuating with seasonal changes in temperature and humidity, or occasional pathogenicity changes due to new gene mutations or exchanges (2–5). It is well-recognized that bacterial co-infection was frequent and critically led to the mortality in influenza virus infection (6, 7). In contrast, previous reports indicated that medical manifestations in cases of dual influenza infection and simple infection are similar (8). Recent ferret model study implied that prior infection antigenically related and unrelated viruses protected from subsequent infection or modified the infection kinetics of the challenge virus (9). The epidemiological and virological study of dual influenza infection cases is of major interest particularly for monitoring emerging influenza strains, which could perpetuate epidemic or pandemic events. However, co-infection with different influenza type/subtype viruses is rare (8, 10), and remain to be fully elucidated due to some limitations of many studies. Co-infections with influenza A subtypes or/and influenza B virus were detected in several surveillance areas regarded as rare events (11). The epidemic of influenza in Anhui Province, China generally exhibited Influenza B, H3N2 and H1N1pdm09 alternating or co-prevalence trend and presented obvious seasonal characteristics, showing single and double peaks, i.e., the peak is bound to occur every winter and spring, while the summer peak occurs in alternate years. In the present study, we described the epidemiological pattern of influenza A and B by surveillance network in Anhui province, eastern China from 2009 to 2014. In addition, we also investigated co-infection of influenza A and B and identified the ssusceptible population.

## Materials and Methods

### Source of Data

Influenza surveillance has been established and developed a surveillance network in Anhui Province of Eastern China since 1990. The network has covered 17 collaborating laboratories of local CDCs and 24 sentinel hospitals until 2009. Based on the guideline of Chinese influenza surveillance (12), the annual surveillance begins at the 14th week (April) of present year and ends at the 13th week (March) of next year, and the collaborating laboratories collected samples from patients with ILI for virological surveillance in sentinel hospitals weekly. Referring physicians at sentinel hospitals were asked to diagnose ILIs according to strict criteria (fever >38°C, cough or sore throat) and to record the number of ILI consultations per day in a fixed format based on age group. These data would be uploaded into Chinese influenza surveillance informatics system by designated hospital staff in hospitals daily. Pharyngeal swab specimens were collected from patients without taking antiviral drugs within 3 days of illness onset. The specimens were transported to the correspondent laboratories in viral transport medium at 4°C for RT-PCR detection and/or viral isolation according to Chinese influenza surveillance guidelines (12). The detection results including weekly type and subtype specific positive rates were input into Chinese influenza surveillance informatics system by collaborating laboratories. In the present study, we collected pharyngeal swab specimens and surveillance data of 5 years from 2009 to 2014 from 17 collaborating laboratories of local CDCs and 24 sentinel hospitals in Anhui Province of China.

### RNA Extraction and RT-PCR

Virus RNA were extracted by RNeasy Mini kit (Qiagen) in pharyngeal swab specimens collected from sentinel hospitals, according to the manufacturers' recommendations. Then we took specific real-time RT-PCR assays for seasonal influenza viruses (H1N1pdm09, H3N2 and influenza B). The nucleic acids of the virus were extracted by GeneRotex Automatic Nucleic acid extractor (TIANLONG Technology CO. LTD). The identification of influenza virus was carried out by QuantStudio Q5 System (Applied Biosystems, Waltham, MA, USA) using AgPath-ID One-step RT-PCR kit (Applied Biosystems, Foster City, CA), the primers and the fluorogenic probes were synthesized according to the national influenza surveillance program (2017 version).

### Isolation of the Virus and Identification of Type/Subtype

The samples of pharyngeal swab were maintained in a viral-transport medium and propagated in MDCK cells for 72 h at 35°C. The supernatant was tested by using hemagglutinin (HA) assay with human “O” type red blood cells, and influenza type and subtype was performed for those with positive hemagglutinin inhibition tests or PCR. The extracted RNA was first tested for the presence of influenza A and B viruses. The nucleic acid showing positive for influenza A/B was further differentiated into subtypes and lineages. Influenza A positive samples were screened for H1pdm and H3 whereas samples showing influenza B positive were tested for B/Yamagata and B/Victoria lineage.

### Haemagglutination Inhibition Assay (HI)

HI was performed as previously described (13). Reference sera were handled with a 1:4 (vol/vol) of receptor disrupting enzyme for 18 h at 37°C and then incubated at 56°C for 30 min. The reference serum was titrated with a 2-fold dilution of PBS (initial dilution of 1:20) and then 4 HAU/25 μl virus was added for the assay.

### Data Analyses

The chi-squared with Fisher's exact test was used to compare dichotomous variables between groups using Graph Prism 5 software (GraphPad Software), and *P* < *0.05* was considered significant.

## Results

### Consultation of ILI in Anhui Province

**Figure 1:**
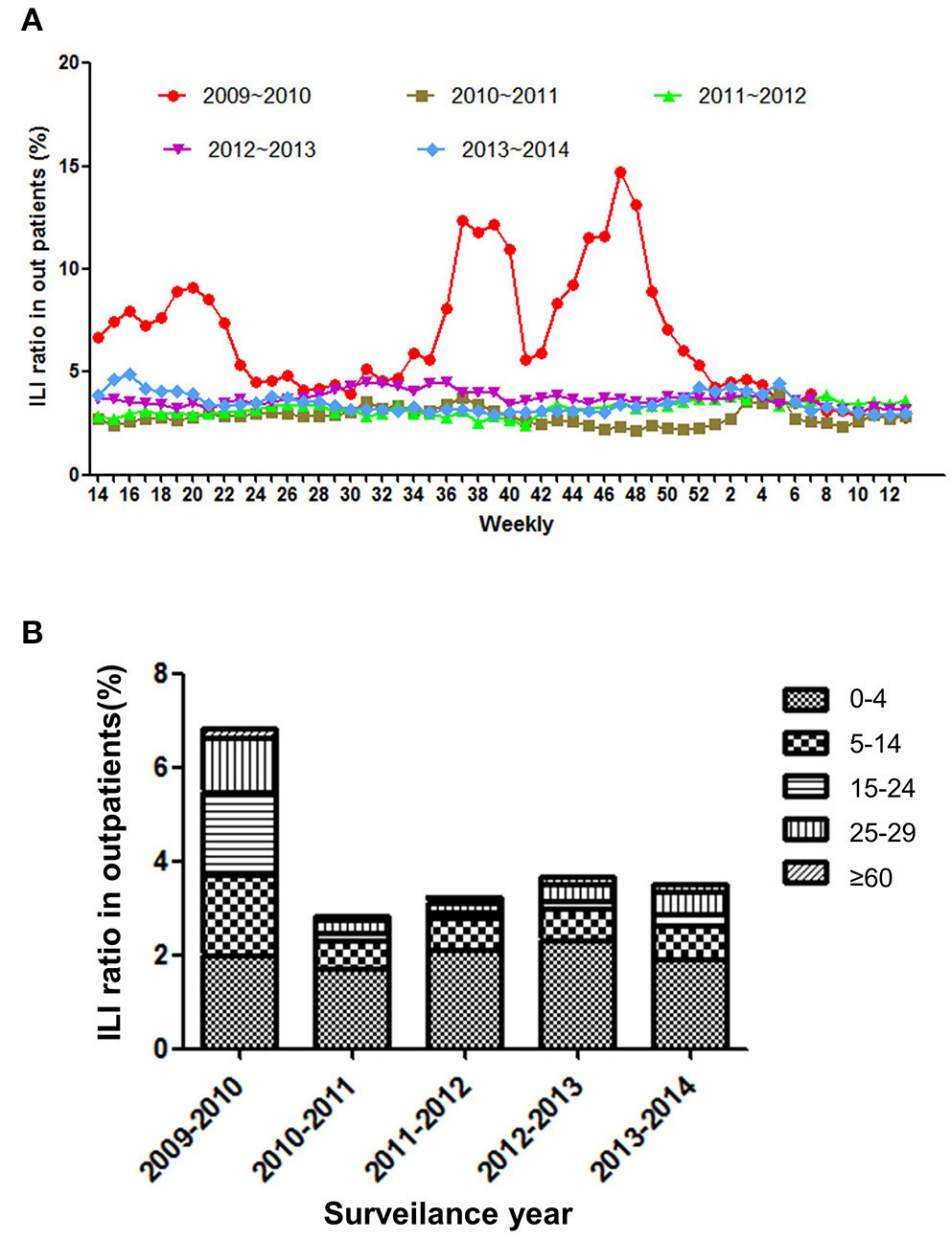
**(A)** The weekly ratio of ILI outpatients from 2009–2014. **(B)** The incidence of ILI outpatients by age group from 2009−2014.

The total number of consultations of ILI was 5,62,551, and the age group percentages by year of surveillance are presented in Supplementary Table 1. The results showed that weekly ILI ratio of outpatients was 3.96 ± 1.9% (95% CI: 3.73–4.2%) for the 5 surveillance years and were the highest in surveillance year of 2009–2010 (6.75 ± 3.05%, 95%CI: 5.72–7.43%). In addition, there were three peaks at 20th, 38th, and 50th weeks during 2009–2010 but not presented in subsequent same seasons (Figure 1A). As is shown in Figure 1B, the youngest patients (<5 years old) presented the highest ILI consultations (2.01 ± 0.22%, 95% CI: 1.74–2.28%). In contrast, the >65 years old population was minimum ILI consultation (0.15 ± 0.05%, 95% CI: 0.08–0.21%) during the influenza epidemic period. To be different in other surveillance years, the ILI consultation of in 2009–2010 present significantly higher level in senior school-aged (15–29 years) population than in school-aged (5–15 years) children *(P* < 0.0001*, OR* = 0.95).

### Epidemic of Influenza Viruses

**Figure 2:**
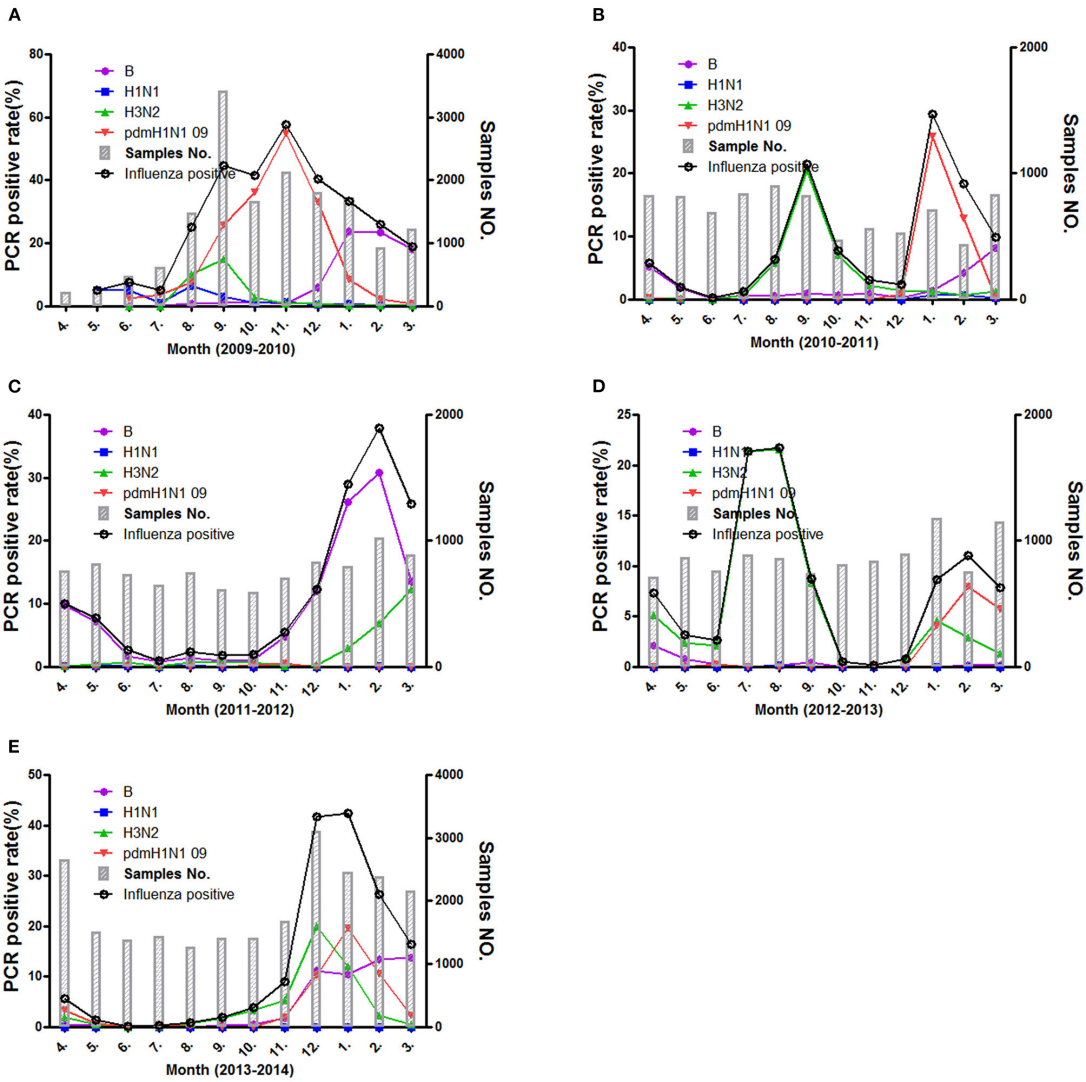
The positive rate of different influenza subtypes during the different surveillance years **(A–E)** from 2009 to 2014.

The epidemic of influenza viruses showed fluctuation in different surveillance year, and the cycling pattern present big difference in these surveillance years. As is shown in Figure 2, the activities of influenza viruses were the strongest in 2009–2010 and kept a high level for long period (July 2009–March 2010) and peaked in November. In addition, three peaks (located in spring, summer, and winter respectively) were present in 2010–2011 and 2012–2013. Both summer peaks were raised by H3N2 influenza virus, whereas only spring and winter peaks presented in 2011–2012 and 2013–2014. In those spring and winter epidemic peaks, influenza A (H1N1pdm09, H3N2) and B exhibited co-circulation. Of note, the novel H7N9 avian influenza virus infection was spotted among ILI patients in 2013 and 2014 by the surveillance system.

### Relative Rate of Influenza Infection

**Figure 3:**
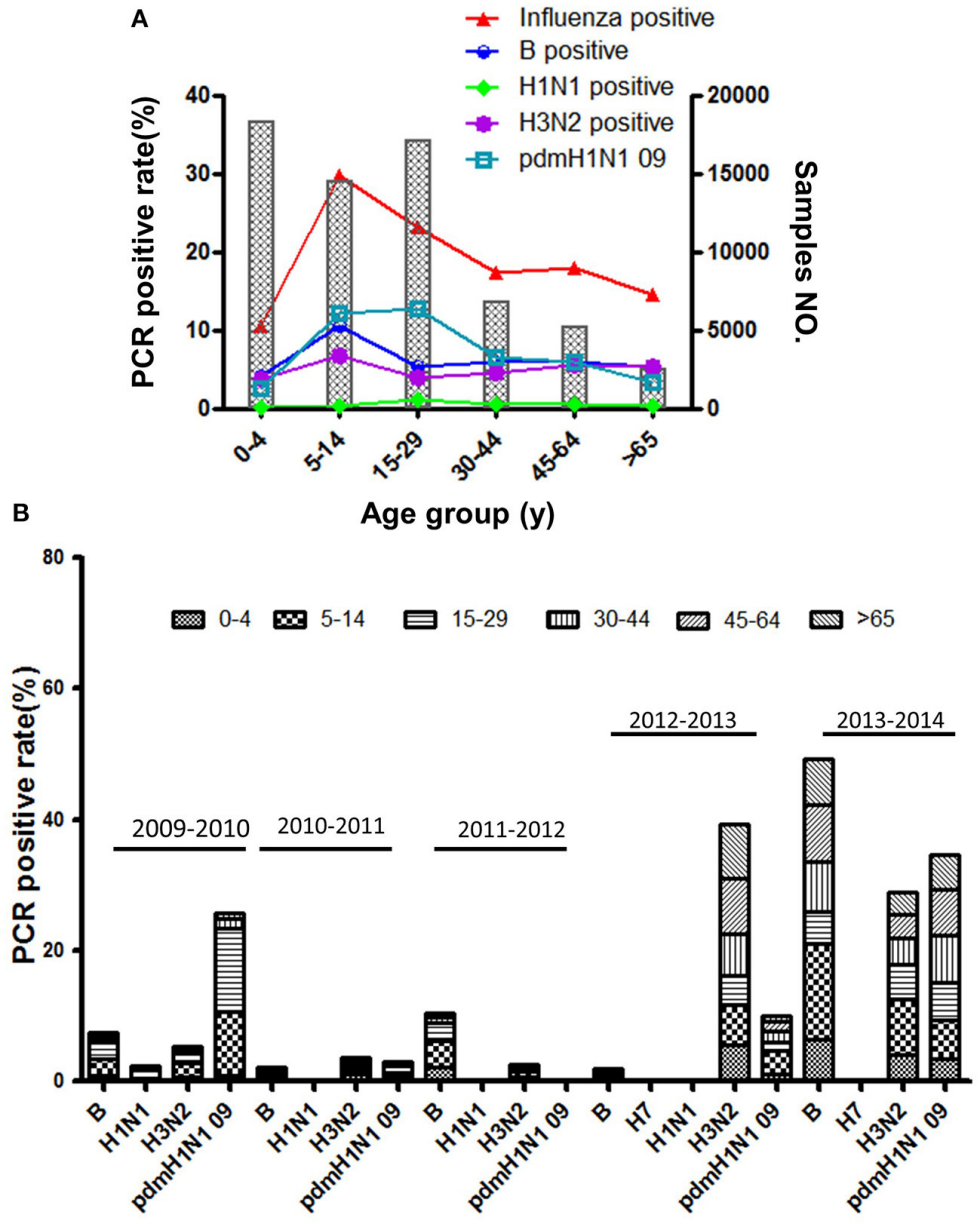
**(A)** The positive rate of different influenza subtypes by age group. **(B)** The age composition ratio of different influenza subtypes from 2009 to 2014.

Based on standard clinical groupings according to potential influenza exposure/risk environments, the 5 years surveillance data showed the influenza consultation difference in different age ILI patients. As is shown in Figure 3A, the influenza infections were the highest in young school-aged patients (24.25 ± 16.52%, 95% CI: 3.74–44.77%), then in senior school-aged people (18.34 ± 16.14%, 95% CI: −1.70–38.38%). To be different with ILI consultation, influenza infection was significantly lowest in the youngest (<5 years old) patients (10.78 ± 5.77%, 95% CI: 3.61–17.95%), and then in oldest (>65 years old) patients (14.36 ± 5.219%, 95% CI: 7.88–20.84%). However, the consultation of senior school-aged patients was significantly decreased (11.29 ± 4.01%, 95% CI: 4.91–17.68%) if we took out pandemic year (2009–2010) data (Supplementary Figure 7). In addition, the H1N1pdm09 virus was the most prevalent influenza virus in ILI patients (Figure 3B), especially in school-aged people (5–29 years old). However, Influenza B and H3N2 virus were more prevalent than H1N1pdm09 if pandemic year (2009–2010) surveillance data was taken out (*P* < 0.001, *OR* = 1.51). More details information associated with total ILI consultations by age group and surveillance year from 2009 to 2014 years were presented in Supplementary Figures 1–7; Supplementary Tables 1, 2.

### Co-infection With Influenza A and B in ILI Patients

**Figure 4:**
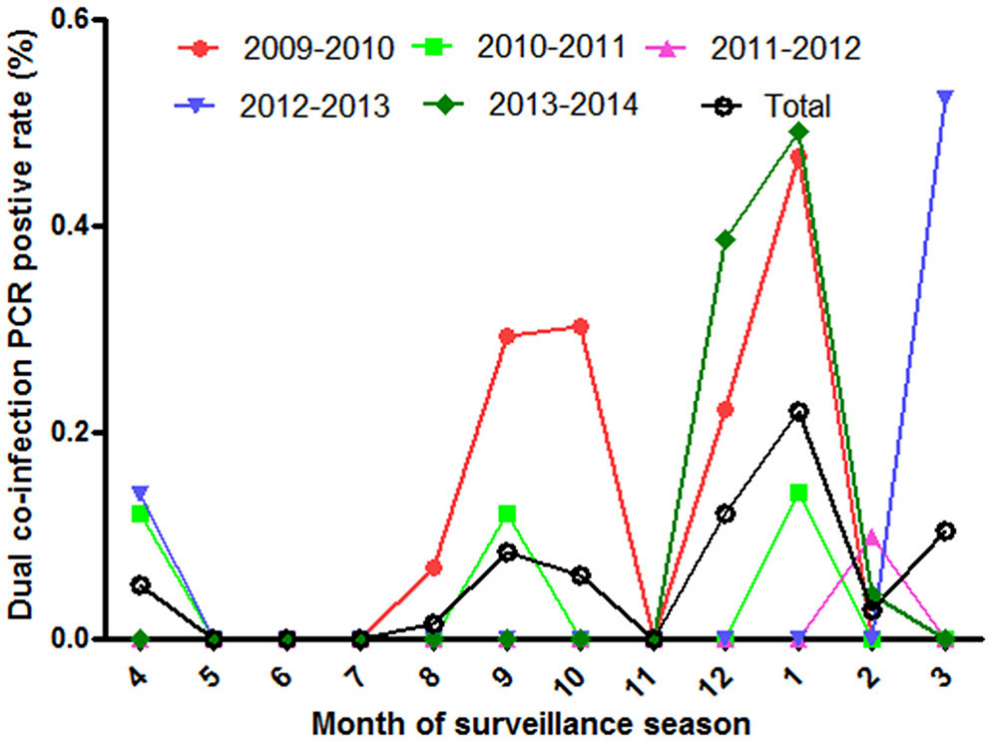
The positive rate of influenza co-infection in different surveillance months from 2009 to 2014.

The co-infection of influenza viruses was detected in ILI patients by PCR during the five surveillance years. The detected rate of co-infection was 0.057% in all collected ILI samples, and 0.58% in influenza PCR positive samples. To be consistent with influenza epidemic season, the positive co-infection peaked in the winter, summer or spring (Figure 4).

To define viral features of co-infection, the available samples were collected for viral isolation. Nine live viruses were isolated from 25 PCR-positive samples, four of which had detectable HI titers for dual viruses, including two H1N1pdm09 combined with Influenza B (Yamagata) and two H1N1pdm09 combined with H3N2 infection, respectively (Table 1). All of these 4 samples were collected from pediatric patients. The remaining five samples were isolated with only a single virus with detectable HI titers.

**Table 1:** Co-infection with influenza A and B in ILI patients enrolled.

**Case ID**	**Gender**	**Age**	**Collecting date of sample**	**Real time RT-PCR**	**Viral isolation (HA titer)**	**HI typing/subtyping (titer)**
1	F	32	2014/2/11	H3 + H1N1pdm09	Neg	ND
2	M	53	2014/1/27	H1N1pdm + B	Pos (16 HAU)	H1N1pdm
3	M	80	2014/1/27	H1N1pdm + B	Neg	ND
4	M	74	2014/1/27	H1N1pdm + B	Neg	ND
5	M	8	2014/1/21	H3 + H1N1pdm	Neg	ND
6	M	1	2014/1/21	H3 + H1N1pdm	Neg	ND
7	M	31	2014/1/22	H3 + H1N1pdm	Neg	ND
8	M	7	2014/1/13	H3 + B	Pos (32 HAU)	H3N2 (1:320), Yamagata (1:1280)
9	F	18	2014/1/6	H3 + H1N1pdm	Neg	ND
10	M	6	2014/1/13	H3 + B	Neg	ND
11	M	41	2014/1/2	H3 + H1N1pdm	Pos (64 HAU)	H1N1pdm (1:1280)
12	F	6	2014/1/2	H3 + B	Pos (32 HAU)	Yamagata (1:1280)
13	F	20	2014/1/1	H1N1pdm + B	Neg	ND
14	M	7	2013/12/25	H3 + H1N1pdm	Pos (16 HAU)	H1N1pdm (1:1280), H3N2(1:320)
15	M	7	2013/12/25	H3 + B	Neg	ND
16	M	7	2013/12/24	H3 + B	Pos (16 HAU)	H3N2(1:320), Yamagata (1:1280)
17	F	7	2013/12/25	H1N1pdm + B	Neg	ND
18	F	38	2013/12/23	H3 + H1N1pdm	Neg	ND
19	M	4	2013/12/23	H3 + H1N1pdm	Pos (16 HAU)	H1N1pdm (1:160), H3N2(1:10)
20	M	78	2013/12/23	H1N1pdm + B	Neg	ND
21	F	3	2013/12/18	H3 + H1N1pdm	Neg	ND
22	M	8	2013/12/18	H3 + B	Pos (128 HAU)	Yamagata (1:1280)
23	F	7	2013/12/16	H3 + B	Neg	ND
24	F	8	2013/12/3	H3 + H1N1pdm	Pos (16 HAU)	H1N1pdm (1:160)
25	M	50	2013/12/9	H1N1pdm + B	Neg	ND

*Data source: The pharyngeal swab specimens and surveillance data of 5 years from 2009 to 2014 were retrieved from 17 collaborating laboratories of local CDCs and 24 sentinel hospitals in Anhui Province of China*.

*F, female; M, male; RT-PCR, Reverse Transcription-Polymerase Chain Reaction; HI, haemagglutination inhibition assay; HA, hemagglutinin; HAU, hemagglutinin unit; Pos, positive; Neg, negative; ND, not detected*.

## Discussion

Effective influenza surveillance systems could help us decipher epidemiology and seasonality of influenza and further optimize possible influenza control strategies. The ILI consultation rates reported by Chinese influenza surveillance network in Anhui province of eastern China showed that ILI affected all aged people through whole year and was remarkably higher in 2009–2010 than in other surveillance years. To be consistent with ILI epidemics, influenza positive rates were the highest in 2009–2010 since H1N1pdm09 outbreak (14, 15). In addition, the data showed that ILI consultation were the highest in children aged <5 years. However, lab detection showed that influenza infections were the most predominant in school aged patients in the surveillance years instead of the youngest patients. In contrast, the most slightly affected people were the youngest patients among the monitored population. The data suggested that other respiratory pathogens may induce youngest ILI patients more frequently than influenza viruses as recent report (16, 17), and school-aged children should be the mainly concerned points for influenza controls in Anhui province China. In addition, the senior school-aged people were more increasingly affected during pandemic year, that is consisted with the previous reports where inpatients in the pandemic season were significantly younger compared with those in the pre-pandemic or post-pandemic seasons (18–20). It may be partially associated with that young adult in the pandemic season were less likely to have been vaccinated (20).

Influenza seasonality in Anhui province China was manifested by seasonal variations in different surveillance years. Except 2009–2010 surveillance year with H1N1 pandemic, two different epidemic patterns were present in the post the 4 surveillance years. Besides the normal winter and spring peaks, a summer peak presented in both 2010–2011 and 2012–2013 surveillance year. Similar recommendations of vaccination have been proposed for China recently (21). Recent data from countries in south and south-eastern Asia also indicate that countries consider vaccination with newly recommended vaccine in May-June prior to seasonal epidemic (22). In addition, the surveillance system presented a high sensitivity on monitoring the activity of the influenza viruses. An H7N9 case who was one of the earliest confirmed cases in mainland China was reported by the system (23, 24). Consequently, the surveillance networks would play a positive role in making evidence-based policy of control and prevention.

In the present study, human co-infections caused by several influenza viruses were detected in the surveillance epidemics. However, live dual viruses were merely detected in a few cases. It means that there was a prepotent virus in the infections with dual influenza viruses associated with viral interference or cross-reactive immune responses (25). Infections with heterosubtypic influenza A viruses presented shortening of virus shedding in previous studies (26). In addition, Cross-reactive epitopes have been found between influenza A and B viruses in the fusion peptide of the HA and enzymatic region of neuraminidase (25). Notably, the possibility of dual virus infection with similar replication level *in vivo* may be more frequent in young patients, that may be associated with host immune response to the infected virus. Previous studies shed a light on the immune response to influenza virus was weaker in the younger children and those without preexisting immunity (27). Theoretically, dual influenza virus infections represent a potential source of multiple viral transmission and constitutes a basis for virus recombination between two human strains or one human and one avian viral strain (28, 29). So, it may be valuable to monitor the potential reassortment due to co-infection in pediatric patients or those without preexisting immunity.

## Conclusion

In the aggregate, we elaborated two different patterns of influenza circulation in different surveillance years in Anhui province, China. In view of school-aged children were the mainly affected patients in ILI associated with influenza viruses, we propose to implement a vaccination program in school-aged population in Anhui province. Influenza B, H3N2 and H1N1pdm09 co-circulated in annual epidemics, and the co-infections with influenza viruses were frequent in epidemic peaks. These data help us implement strategies to select vaccine formulations and monitor potential recombinant viruses in surveillance programs.

## Data Availability Statement

Influenza surveillance data used and analyzed in the current study are available from the corresponding authors on reasonable request.

## Author Contributions

R-BG and H-FP conceptualized the review. Literature search was performed by J-BW and X-EH. Supervision was performed by LG and JL. The first draft and editing of the manuscript were performed by JH, SH, and YC. Visualization/data presentation were performed by J-LY, Q-QC, and LH. All authors commented on previous versions of the manuscript, read, and approved the final manuscript.

## Funding

This work was supported by the Scientific Research Projects of Health Commission of Anhui Province in 2021 (grant number: AHWJ2021a030).

## Conflict of Interest

The authors declare that the research was conducted in the absence of any commercial or financial relationships that could be construed as a potential conflict of interest.

## Publisher's Note

All claims expressed in this article are solely those of the authors and do not necessarily represent those of their affiliated organizations, or those of the publisher, the editors and the reviewers. Any product that may be evaluated in this article, or claim that may be made by its manufacturer, is not guaranteed or endorsed by the publisher.

## Abbreviations

ILI: influenza-like illness
HI: haemagglutination inhibition assay
HA: hemagglutinin.

## References

[1] Iuliano AD Roguski KM Chang HH Muscatello DJ Palekar R Tempia S. Estimates of global seasonal influenza-associated respiratory mortality: a modelling study. Lancet. (2018) 391:1285–300. 10.1016/S0140-6736(17)33293-2 29248255 PMC5935243

[2] Chadha MS Potdar VA Saha S Koul PA Broor S Dar L. Dynamics of influenza seasonality at sub-regional levels in India and implications for vaccination timing. PLoS ONE. (2015) 10:e0124122. 10.1371/journal.pone.0124122 25938466 PMC4418715

[3] Linde A Rotzen-Ostlund M Zweygberg-Wirgart B Rubinova S Brytting M. Does viral interference affect spread of influenza? Euro Surveill. (2009) 14:19354. 10.2807/ese.14.40.19354-en 19822124

[4] Greenbaum BD Li OT Poon LL Levine AJ Rabadan R. Viral reassortment as an information exchange between viral segments. Proc Natl Acad Sci USA. (2012) 109:3341–6. 10.1073/pnas.1113300109 22331898 PMC3295259

[5] Lam TT Hon CC Pybus OG Kosakovsky Pond SL Wong RT Yip CW. Evolutionary and transmission dynamics of reassortant H5N1 influenza virus in Indonesia. PLoS Pathog. (2008) 4:e1000130. 10.1371/journal.ppat.1000130 18725937 PMC2515348

[6] Cauley LS Vella AT. Why is coinfection with influenza virus and bacteria so difficult to control? Discov Med. (2015) 19:33–40. 25636959 PMC4313126

[7] Ackerman E Longini IM Jr Seaholm SK Hedin AS. Simulation of mechanisms of viral interference in influenza. Int J Epidemiol. (1990) 19:444–54. 10.1093/ije/19.2.444 2376460

[8] Almajhdi FN Ali G. Report on influenza A and B viruses: their coinfection in a Saudi leukemia patient. BioMed Res Int. (2013) 2013:290609. 10.1155/2013/290609 24078911 PMC3775437

[9] Laurie KL Guarnaccia TA Carolan LA Yan AW Aban M Petrie S. Interval between infections and viral hierarchies are determinants of viral interference following influenza virus infection in a ferret model. J Infect Dis. (2015) 212:1701–10. 10.1093/infdis/jiv260 25943206 PMC4633756

[10] Falchi A Arena C Andreoletti L Jacques J Leveque N Blanchon T. Dual infections by influenza A/H3N2 and B viruses and by influenza A/H3N2 and A/H1N1 viruses during winter 2007, Corsica Island, France. J Clin Virol. (2008) 41:148–51. 10.1016/j.jcv.2007.11.003 18069055

[11] World Health Organization. Influenza Update. (2015). Available online at: https://www.who.int/en/news-room/fact-sheets/detail/influenza-(seasonal). (accessed May 15, 2015).

[12] National Health Commission of the People's Republic of China. Guideline of Chinese Influenza Surveillance. (2017). Available online at: http://www.nhc.gov.cn/wjw/index.shtml (accessed March 30, 2017).

[13] Xu C Bai T Iuliano AD Wang M Yang L Wen L. The seroprevalence of pandemic influenza H1N1 (2009) virus in China. PLoS ONE. (2011) 6:e17919. 10.1371/journal.pone.0017919 21533034 PMC3080876

[14] Wei M Yan Z Wang C Liu W Cao W. Eight-hospital based influenza like illness surveillance from April, 2009 to March, 2011 in China. Influenza Other Respir Viruses. (2013) 7:997–8. 10.1111/irv.12064 23216804 PMC4634270

[15] Peng Y Xu Y Zhu M Yu H Nie S Yan W. Chinese urban-rural disparity in pandemic (H1N1) 2009 vaccination coverage rate and associated determinants: a cross-sectional telephone survey. Public Health. (2013) 127:930–7. 10.1016/j.puhe.2013.06.007 24139202

[16] Lekana-Douki SE Nkoghe D Drosten C Ngoungou EB Drexler JF Leroy EM. Viral etiology and seasonality of influenza-like illness in Gabon, March 2010 to June 2011. BMC Infect Dis. (2014) 14:373. 10.1186/1471-2334-14-373 25000832 PMC4107952

[17] Kocik J Niemcewicz M Winnicka I Michalski A Bielawska-Drozd A Kolodziej M. Diversity of influenza-like illness etiology in Polish Armed Forces in influenza epidemic season. Acta Biochim Pol. (2014) 61:489–94. 10.18388/abp.2014_1869 25195140

[18] Reed C Chaves SS Perez A D'Mello T Daily Kirley P Aragon D. Complications among adults hospitalized with influenza: a comparison of seasonal influenza and the 2009 H1N1 pandemic. Clin Infect Dis. (2014) 59:166–74. 10.1093/cid/ciu285 24785230 PMC7314251

[19] Dawood FS Iuliano AD Reed C Meltzer MI Shay DK Cheng PY. Estimated global mortality associated with the first 12 months of 2009 pandemic influenza A H1N1 virus circulation: a modelling study. Lancet Infect Dis. (2012) 12:687–95. 10.1016/S1473-3099(12)70121-4 22738893

[20] Mitchell R Taylor G McGeer A Frenette C Suh KN Wong A. Understanding the burden of influenza infection among adults in Canadian hospitals: a comparison of the 2009-2010 pandemic season with the prepandemic and postpandemic seasons. Am J Infect Control. (2013) 41:1032–7. 10.1016/j.ajic.2013.06.008 24176768

[21] Yu H Alonso WJ Feng L Tan Y Shu Y Yang W. Characterization of regional influenza seasonality patterns in China and implications for vaccination strategies: spatio-temporal modeling of surveillance data. PLoS Med. (2013) 10:e1001552. 10.1371/journal.pmed.1001552 24348203 PMC3864611

[22] Saha S Chadha M Al Mamun A Rahman M Sturm-Ramirez K Chittaganpitch M. Influenza seasonality and vaccination timing in tropical and subtropical areas of southern and south-eastern Asia. Bull World Health Org. (2014) 92:318–30. 10.2471/BLT.13.124412 24839321 PMC4007122

[23] Gao R Cao B Hu Y Feng Z Wang D Hu W. Human infection with a novel avian-origin influenza A (H7N9) virus. N Engl J Med. (2013) 368:1888–97. 10.1056/NEJMoa1304459 23577628

[24] Li Q Zhou L Zhou M Chen Z Li F Wu H. Epidemiology of human infections with avian influenza A(H7N9) virus in China. N Engl J Med. (2014) 370:520–32. 10.1056/NEJMoa1304617 23614499 PMC6652192

[25] Terajima M Babon JA Co MD Ennis FA. Cross-reactive human B cell and T cell epitopes between influenza A and B viruses. Virol J. (2013) 10:244. 10.1186/1743-422X-10-244 23886073 PMC3726517

[26] Laurie KL Carolan LA Middleton D Lowther S Kelso A Barr IG. Multiple infections with seasonal influenza A virus induce cross-protective immunity against A(H1N1) pandemic influenza virus in a ferret model. J Infect Dis. (2010) 202:1011–20. 10.1086/656188 20715930

[27] Mugitani A Ito K Irie S Eto T Ishibashi M Ohfuji S. Immunogenicity of the trivalent inactivated influenza vaccine in young children less than 4 years of age, with a focus on age and baseline antibodies. Clin Vaccine Immunol. (2014) 21:1253–60. 10.1128/CVI.00200-14 24990904 PMC4178574

[28] Sampath R Russell KL Massire C Eshoo MW Harpin V Blyn LB. Global surveillance of emerging Influenza virus genotypes by mass spectrometry. PLoS ONE. (2007) 2:e489. 10.1371/journal.pone.0000489 17534439 PMC1876795

[29] Tramuto F Maida CM Magliozzo F Amodio E Vitale F. Occurrence of a case of influenza A(H1N1)pdm09 and B co-infection during the epidemic season 2012-2013. Infect Genet Evol. (2014) 23:95–8. 10.1016/j.meegid.2014.01.032 24518691

