# Supplementary material for: The Epidemiological Pattern and Co-infection of Influenza A and B by Surveillance Network From 2009 to 2014 in Anhui Province, China
Source: Front Public Health. 2022 Feb 24;10:825645. doi: 10.3389/fpubh.2022.825645 (PMC8907529; doi:10.3389/fpubh.2022.825645)
Supplement: Supplementary file 1 [file Data_Sheet_1.docx]

**Supplymentary figures 1-6**

**Total number of consultations of ILI by age group and surveillance year from 2009 to 2014 years**

**Figure 1**

**
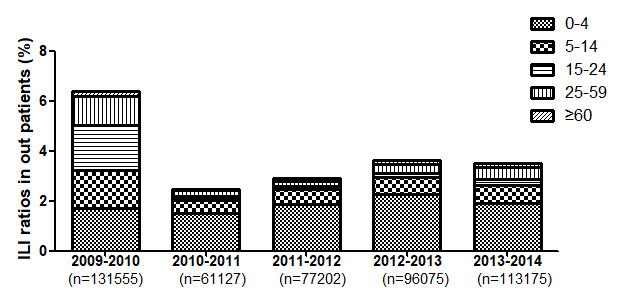
**

**Figure 2**


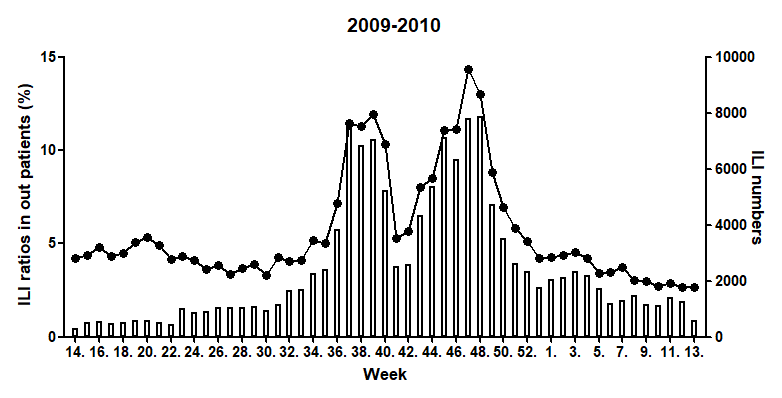


**Figure 3**


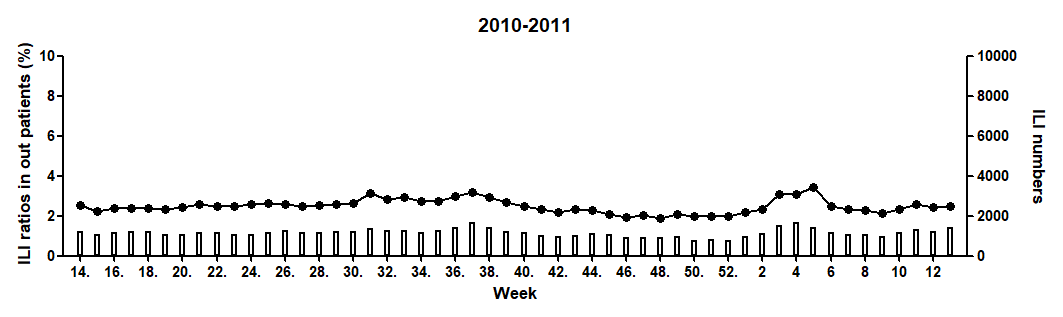


**Figure 4**


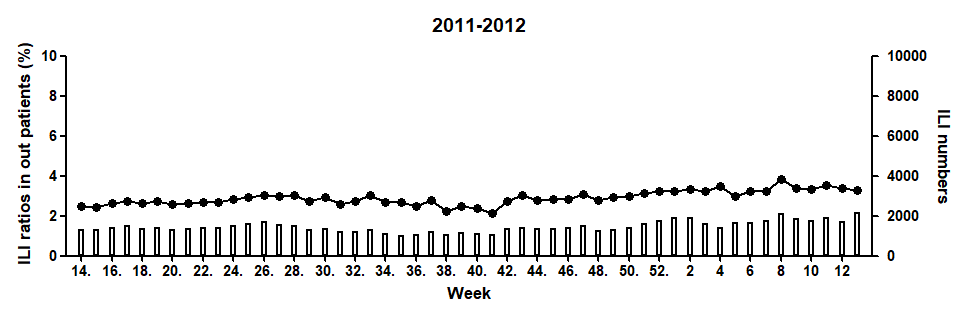


**Figure 5**


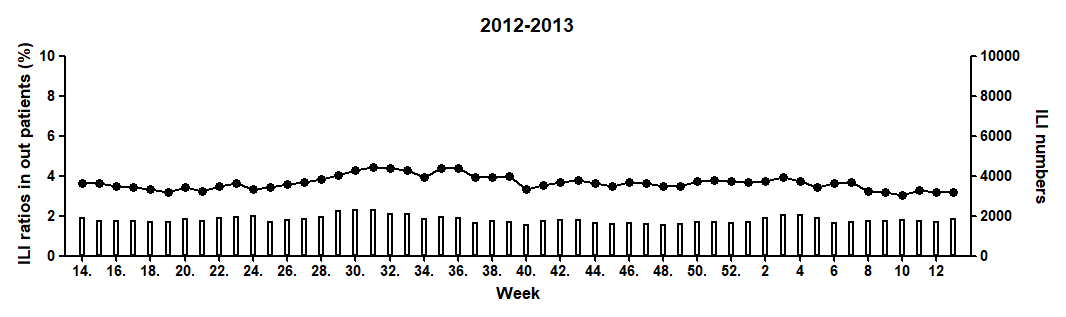


**Figure 6**


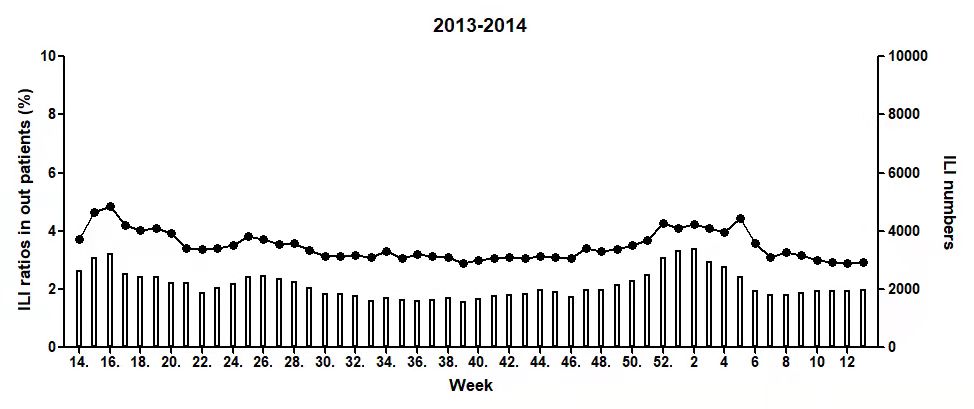


**Figure 7 The subgroup distribution of ILI from 2009 to 2014 years**


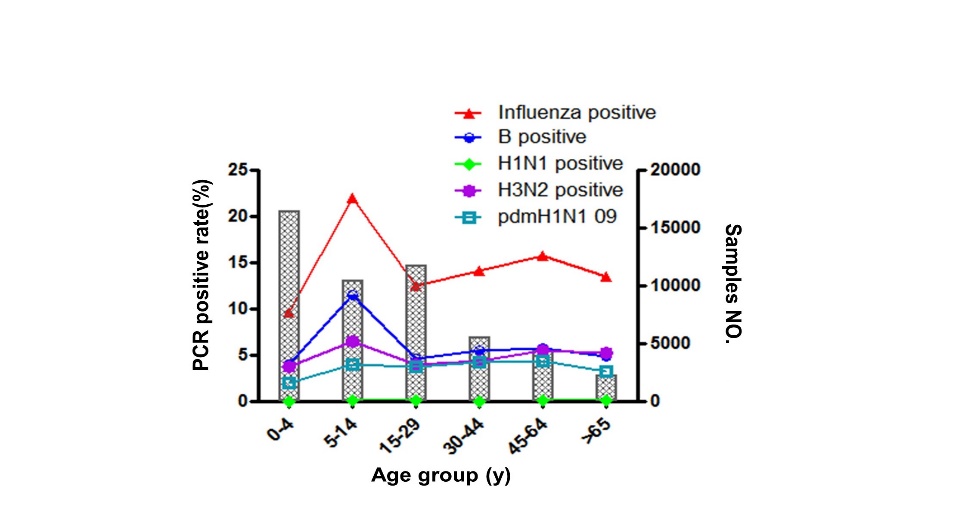


**Supplementary tables 1-2**  Table 1 Age distribution of influenza-like illnesses from 2009 to 2014

| Year | Age | | | | |  |
| --- | --- | --- | --- | --- | --- | --- |
|  | 0-4 years (%) | 5-14 years (%) | 15-24 years (%) | 25-59 years (%) | ≥60 years (%) | N |
| 2009-2010 | 35493 (27.0) | 31027 (23.6) | 37380(28.4) | 23865(18.1) | 3790 (2.9) | 131555 |
| 2010-2011 | 37033 (60.6) | 12630(20.7) | 3625(5.9) | 6278(10.3) | 1561 (2.6) | 61127 |
| 2011-2012 | 49298 (63.9) | 16161 (20.9) | 3219(4.2) | 5641 (7.3) | 2883(3.7) | 77202 |
| 2012-2013 | 60194 (62.7) | 17615 (18.3) | 3941 (4.1) | 9274(9.7) | 5051 (5.3) | 96075 |
| 2013-2014 | 62064 (54.8) | 22829(20.2) | 7941 (7.0) | 14916(13.2) | 5425 (4.8) | 113175 |
| Overall | 244082(50.9) | 100262(20.9) | 56106 (11.7) | 59974 (12.5) | 18710(3.9) | 479134 |

Table 2 Subtypes distribution of influenza-like illnesses from 2009 to 2014

| Subtypes | H3N2  N (%) | H1N1pdm09  N (%) | Influenza B  N (%) | Hybrid  N (%) | Others  N (%) | Total  N |
| --- | --- | --- | --- | --- | --- | --- |
| 2009-2010 | 729(13.9) | 3377(64.4) | 186(3.5) | 20(0.4) | 935(17.8) | 5247 |
| 2010-2011 | 298(37.4) | 241(30.2) | 176(22.1) | 10(1.3) | 72(9.0) | 797 |
| 2011-2012 | 226(19.9) | 6(0.5) | 864(76.1) | 1(0.1) | 39(3.4) | 1136 |
| 2012-2013 | 594(68.5) | 167(19.3) | 29(3.3) | 2(0.2) | 75(8.7) | 867 |
| 2013-2014 | 1053(31.9) | 1135(34.4) | 1071(32.5) | 18(0.5) | 22(0.7) | 3299 |
| Overall | 2900(25.6) | 4926(43.4) | 2326(20.5) | 51(0.4) | 1143(10.1) | 11346 |
